# Allergic diseases of the skin and drug allergies – 2030. Validation of cephalosporin skin test for prediciting immediate hypersensitivity: interim analysis

**DOI:** 10.1186/1939-4551-6-S1-P116

**Published:** 2013-04-23

**Authors:** Sun-Young Yoon, So Young Park, Sujeong Kim, Hyouk-Soo Kwon, Yun-Jeong Bae, Jaehyung Roh, You Sook Cho, Tae-Bum Kim, Hee-Bom Moon

**Affiliations:** 1 Division of Allergy and Clinical Immunology, Department of Internal Medicine, Asan Medical Center, University of Ulsan College of Medicine, Seoul, South Korea; 2 Health Medicine, Health Screening and Promotion Center, Asan Medical Center, Seoul, South Korea; 3 Bonghwa Hospital, Gyungsangbukdo, South Korea; 4 Asian Medical Center, College of Medicine Ulsan University, South Korea

## Background

Cephalosporin is one of the most commonly used beta-lactam antibiotics globally, and also a major offending agent of drug hypersensitivity along with penicillin. Cephalosporin skin test has been widely used in most hospitals in Korea. However, the validity of this test for prediction of immediate hypersensitivity has still not been well studied. Therefore, we conducted this study to determine the predictive validity of cephalosporin skin test prior to administration.

## Methods

We prospectively conducted intradermal skin test with selected 1^st^, 2^nd^, and 3rd generation cephalosporins: ceftazol, cefotetan or cefamandol, ceftriaxone or cefotaxime or flomoxef, respectively. After skin test, one of the tested cephalosporins was intravenously administered for preoperative prevention of infection under careful observation, regardless of the skin test results.

## Results

We recruited 1,125 patients who needed the use of preoperative cephalosporins. Eighty five patients (7.5%) showed positive skin test to at least one cephalosporin. However, none of these patients showed immediate hypersensitivity reactions. Two patients who showed generalized urticaria and itching sense had negative skin test. The test showed sensitivity of 0%, specificity of 92.4%, negative predictive value of 99.8%, and positive predictive value 0%.

## Conclusions

Routine skin test of cephalosporin prior to its administration is not valid for predicting immediate hypersensitivity with low sensitivity and positive predictive value. This study is currently ongoing for enrollment of larger study group.

